# Calreticulin surface presentation: a signal for natural killer cells to attack

**DOI:** 10.1038/s41392-023-01551-z

**Published:** 2023-08-07

**Authors:** Jasmine P. Castellanos, Joseph C. Genereux

**Affiliations:** 1 Microbiology Graduate Program, University of California, Riverside, CA 92521 USA; 2 Department of Chemistry, University of California, Riverside, CA 92521 USA

**Keywords:** Tumour immunology, Innate immunity

In a recent article published in *Nature*, Sen Santara et al. report a general mechanism that allows endoplasmic reticulum (ER) stress, a common response to many cellular dysfunctions, to signal to natural killer (NK) cells to destroy the stressed cell (Fig. 1).^1^ This mechanism suggests that targeting this signaling pathway might serve as a common therapeutic approach to treat disparate diseases, including cancers and infections.

**Fig. 1:**
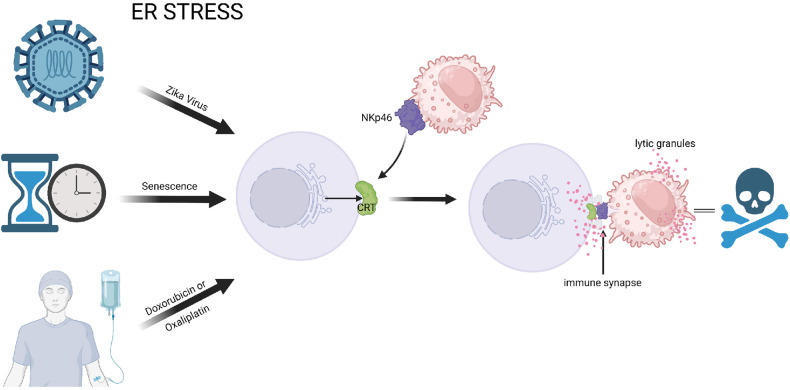
ER stress is connected to diverse cellular dysfunctions, including infection by the Zika virus, senescence, and some chemotherapeutic treatments. When these cells undergo ER stress, the stress enables non-canonical secretion of ER chaperones to the cell surface, including CRT. CRT at the cell surface recruits NK cells through NKp46, with consequent cytotoxicity. Created with BioRender.com

The innate immune system recognizes molecular signatures that indicate threats to the organism, and transduces these signatures into an immune response to counter these threats. As part of the innate immune system, NK cells constitute a lymphoid immune cell type that kills infected or cancerous cells on the basis of their presentation of specific ligands or markers on the surface of abnormal cells. Although about two dozen activating receptors have been described for NK cells, the targets that mediate the recognition of cells undergoing diverse stresses have not been well-characterized.

The study by Santara et al. grew out of their initial observation that NK cells kill cells infected with ER stress-inducing viruses, such as ZIKV, but not viruses that do not induce ER stress, such as CMV and HSV-2. They demonstrated that NK release of lytic granules is specifically driven by ER stress, and in particular mediated by ATF6 and PERK activation downstream of ER stress. Through the use of blocking antibodies targeting NK receptors, NKp46 was determined to mediate NK killing of ZIKV-infected cells. With the receptor identified, they used affinity purification and mass spectrometry to determine that external presentation on the cell surface of calreticulin (CRT), an ER lectin chaperone, is necessary and sufficient to activate NKp46 and thus NK cytotoxic activity. This is distinct from the previously reported role of cell-surface CRT (ecto-CRT) in diverse cancer cells activating phagocytosis by macrophages.^2^ The interaction was further confirmed through careful biophysical studies that determined the specific regions driving CRT-NKp46 association. The PERK and ATF6 dependence is consistent with the specific regulation of CRT expression by ATF6 activation^3^ and the stimulation of ER chaperone externalization by PERK activity.^4^

In addition to viral infection, ER stress can be induced by several chemotherapeutics that are associated with immunogenic cell death (ICD), such as oxaliplatin and doxorubicin. The authors demonstrated that oxaliplatin, but not cisplatin, treatment of B16-F10 melanoma cells induces ecto-CRT, leading to killing by NK cells in a NKp46 mediated manner. Critically, mice xenografts with B16-F10 tumors were only responsive to doxorubicin treatment when NKp46 is expressed, suggesting that this signaling pathway is necessary for the mechanism of action of these chemotherapeutics. Another pathway by which chemotherapeutics can decrease tumor size is through the induction of senescence. Senescence is a significant mechanism of tumor suppression, both basally and following chemotherapeutic treatment, and has been reported to be driven in part by the RIDD pathway downstream of ER stress. The authors induced senescence in A549 human lung cancer cells with the chemotherapeutics trametinib and palbociclib, which also induce ER stress. The treated cells were far more effectively targeted by NK cells, and again in a NKp46-dependent manner. Similar results were observed for mouse xenografts from KP lung cancer cells.

To better characterize the downstream signaling events associated with ecto-CRT expression, the authors incorporated a GPI-tag on CRT that forces its ectopic cell surface expression in the absence of ER stress. These cells readily form immune synapses with NK cells in a NKp46-dependent manner with consequent cytotoxicity. In mice lacking NKp46, these engineered cells demonstrate increased cell surface, migration and invasion, and colony formation, suggesting that ecto-CRT contributes to the tendency of cells to become cancerous. In mice with NKp46, by contrast, ecto-CRT failed to drive metastasis and indeed NK cells were associated with an increase in tumor response markers, as compared to NKp64-deficient mice.

These findings suggest that the NKp46-ecto-CRT interaction plays a significant role in tumor immune responses and could be targeted for therapeutic interventions. They also add to our emerging understanding of how ER chaperones, traditionally associated with their roles within the ER, can impact immune recognition and tumor immunity. Previous studies have shown that the surface exposure of chaperones like GRP78^4^ and calreticulin^5^ in cancer cells promotes cancer cell survival, metastasis, and immune evasion, making it an attractive target for therapeutic interventions. By identifying and targeting these cells with high cell surface ER chaperone levels, it may be possible to attack the cancer cells with the most significant malignant risk specifically. Furthermore, interventions that either target ecto-CRT directly or enhance its recognition by NK cells could be used broadly against cellular dysfunctions associated with ER stress.

## References

[1] Sen Santara S (2023). The NK cell receptor NKp46 recognizes ecto-calreticulin on ER-stressed cells. Nature.

[2] Chao MP (2010). Calreticulin is the dominant pro-phagocytic signal on multiple human cancers and is counterbalanced by CD47. Sci. Transl. Med..

[3] Shoulders MD (2013). Stress-independent activation of XBP1s and/or ATF6 reveals three functionally diverse ER proteostasis environments. Cell Rep..

[4] Van Krieken R, Tsai Y-L, Carlos AJ, Ha DP, Lee AS (2021). ER residential chaperone GRP78 unconventionally relocalizes to the cell surface via endosomal transport. Cell. Mol. Life Sci..

[5] Zhang, M. et al. Calreticulin as a marker and therapeutic target for cancer. *Clin. Exp. Med*. 10.1007/s10238-022-00937-7 (2022). 10.1007/s10238-022-00937-7 36335525

